# Ozone Treatment Is Insufficient to Inactivate SARS-CoV-2 Surrogate under Field Conditions

**DOI:** 10.3390/antiox10091480

**Published:** 2021-09-16

**Authors:** Natalia Mazur-Panasiuk, Pawel Botwina, Adrian Kutaj, Damian Woszczyna, Krzysztof Pyrc

**Affiliations:** 1 Virogenetics Laboratory of Virology, Malopolska Centre of Biotechnology, Jagiellonian University, Gronostajowa 7A, 30-387 Krakow, Poland; natalia.mazur-panasiuk@uj.edu.pl (N.M.-P.); pawel.botwina@doctoral.uj.edu.pl (P.B.); 2 Microbiology Department, Faculty of Biochemistry, Biophysics and Biotechnology, Jagiellonian University, Gronostajowa 7, 30-387 Krakow, Poland; 3 Rescue and Fire Fighting Services no. 6, Regional Headquarters of the State Fire Service in Krakow, Aleksandry 2, 30-837 Krakow, Poland; adrian.kutaj93@gmail.com (A.K.); damian.woszczyna@psp.krakow.pl (D.W.)

**Keywords:** SARS-CoV-2, disinfection, ozone, hydrogen peroxide, quantitative carrier test

## Abstract

COVID-19 caused by SARS-CoV-2 caused a worldwide crisis, highlighting the importance of preventive measures in infectious diseases control. SARS-CoV-2 can remain infectious on surfaces for up to several weeks; therefore, proper disinfection is required to mitigate the risk of indirect virus spreading. Gaseous ozone treatment has received particular attention as an easily accessible disinfection tool. In this study, we evaluated the virucidal effectiveness of gaseous ozone treatment (>7.3 ppm, 2 h) on murine hepatitis virus (MHV)-contaminated stainless-steel surface and PBS-suspended virus under field conditions at ambient (21.8%) and high (49.8–54.2%) relative humidity. Surficial virus was soiled with 0.3 g/L of BSA. Parallelly, a half-hour vaporization with 7.3% hydrogen peroxide was performed on contaminated carriers. The obtained results showed that gaseous ozone, whilst quite effective against suspended virus, was insufficient in sanitizing coronavirus contaminated surfaces, especially under low RH. Increased humidity created more favorable conditions for MHV inactivation, resulting in 2.1 log titre reduction. Vaporization with 7.3% hydrogen peroxide presented much better virucidal performance than ozonation in a similar experimental setup, indicating that its application may be more advantageous regarding gaseous disinfection of surfaces contaminated with other coronaviruses, including SARS-CoV-2.

## 1. Introduction

In recent months, severe acute respiratory syndrome coronavirus 2 (SARS-CoV-2) has spread worldwide, causing a global health crisis. SARS-CoV-2 is transmitted mainly via respiratory aerosols and droplets generated during breathing, speaking, coughing, and sneezing [1]. The risk of infection in an outdoor environment is considered low [2]. On the contrary, aerosol transmission in poorly ventilated interiors is reported to play a significant role in airborne transmission [3]. Furthermore, the virus-containing droplets quickly settle onto nearby surfaces, highlighting the need for frequent disinfection of spaces with prolonged contact with infected individuals [4,5]. Early reports showed that SARS-CoV-2 loses its infectivity faster on porous than non-porous surfaces, where it can persist up to 14 days [6,7]. More recently, it has been demonstrated that the pathogen persists even up to 21 days on smooth surfaces such as plastic [8]. With respect to indirect transmission, one of the most spectacular cases was documented in Beijing, where contaminated cold-chain food was demonstrated as the source of the disease outbreak in seafood market workers [9]. Although few indirect transmission cases have been documented, contact with contaminated fomites is still considered a possible route for disease spreading [7,10,11,12].

Lipid bi-layered envelope makes coronaviruses an easy target for common disinfectants based on alcohols, sodium hypochlorite, potassium peroxymonosulfate, quaternary ammonium salts, aldehydes, and hydrogen peroxide [13,14]. Although effective, the chemical disinfection approach is inappropriate for sanitizing inaccessible surfaces and hard-to-reach places. For this reason, fumigation with gaseous formaldehyde, vaporized hydrogen peroxide, or ozone become widely used decontamination tools for interiors, cars, and objects, e.g., furniture and personal protective equipment (PPE) [15].

Ozone is an excellent biocidal agent due to its strong oxidizing properties, and its effectiveness was confirmed on bacteria, fungi, and viruses. Thus, it is commonly applied for water treatment, food preservation, and other purposes [16,17,18]. Ozone has a high solubility in water, but it decomposes rapidly due to its instability, resulting in the by production of free radicals [19]. Therefore, the ozonized water has been shown to inactivate numerous RNA and DNA viruses, including SARS-CoV-2 [20,21]. Due to shortages of PPE at the beginning of the COVID-19 pandemic, gaseous ozone disinfection becomes an attractive tool, providing further use of disposable protective equipment [8,22,23]. Moreover, the low cost of ozone generators makes them easily accessible; therefore, they began to be widely applied to decontaminate many closed spaces, such as offices, classrooms, churches, hospitals, and public utility buildings. Up to this point, several studies were committed to investigating the efficiency of gaseous ozone treatment in relation to SARS-CoV-2 or its surrogates [15,16,22,24,25,26,27,28,29].

Biocidal activity of various disinfection methods may be confirmed by international standards, some of which are developed with respect either to medical, veterinary, or domestic areas. In the European Union, the virucidal activity of gaseous disinfection is regulated by EN 17272:2020: “Chemical disinfectants and antiseptics-Methods of airborne room disinfection by automated process-Determination of bactericidal, mycobactericidal, sporicidal, fungicidal, yeasticidal, virucidal and phagocidal activities”. Regarding viruses in medical areas, the standard implements quantitative carrier tests (QCT) where selected pathogens suspended in the protein-containing buffer are dried on sterile surfaces and subjected to airborne disinfection. The disinfection is considered effective when at least four orders of magnitude reduction in virus titre is achieved compared to the amount of pathogen recovered from untreated carriers [30]. 

The aim of this study was to investigate the effectiveness of gaseous ozone generated by dielectric barrier discharge and hydrogen peroxide fumigation conducted under field conditions. The experimental design was inspired by the BS-EN 17272:2020 standard and QCTs. Moreover, PBS-suspended virus was parallelly subjected to ozonation. The study was carried out in a real-world setting; thus, the application of human coronaviruses was not possible due to biosafety issues. Hence, the other nonpathogenic for humans betacoronavirus, i.e., murine hepatitis virus (MHV), was selected as the surrogate [31,32]. MHV is also a betacoronavirus, sharing genetic and morphological similarity with SARS-CoV-2, and it has been repeatedly used as a SARS-CoV-2 surrogate in other studies [33,34,35].

## 2. Materials and Methods

### 2.1. Cells and Viruses

MHV A59 strain was purchased from ATCC (ATCC^®^ VR-764™). The virus was propagated in murine L cells (LR7) [33]. The cells were maintained in Dulbecco’s Modified Eagle’s Medium (DMEM), supplemented by 10% fetal bovine serum (FBS), 1% penicillin-streptomycin, and 1% geneticin (G418 sulfate), at 37 °C in a humidified 5% CO2 enriched atmosphere. All cell culture media and supplements were purchased at Gibco, Thermofisher Scientific, Waltham, MA, USA. In order to prepare virus stocks, LR7 cell cultures at 90% confluence were inoculated with 10^3^ TCID_50_/_mL_ of MHV, followed by incubation until the evident cytopathic effect was developed. On the final day, the virus was released by freezing-thawing methods, and the suspension was cleared by centrifugation (2000× *g*; 10 min, 4 °C), aliquoted, and stored at −80 °C until further use.

### 2.2. Virus–PBS Solution

Phosphate-buffered saline (PBS) was purchased at Gibco, Thermofisher Scientific, Waltham, MA, USA. The virus stock was 100× diluted in PBS; subsequently, 1 mL of the obtained solution was added into 30 mm Petri dishes and immediately subjected to ozone treatment.

### 2.3. Virus–Protein Solution

The study was performed in low-soiling conditions according to BS-EN 17272:2020 standard. The water solution of bovine serum albumin (3 g/L; BSA, Sigma Aldrich, St. Louis, MO, USA) was prepared and filtered through a 0.22 μm syringe filter on the day of the experiment. Immediately before setting up the study, the solution was 10× diluted with virus stock in order to obtain a working BSA solution (0.3 g/L).

### 2.4. Carriers

Non-porous, stainless-steel discs of φ = 22 mm were prepared by a CNC milling machine thanks to the courtesy of State Fire Service workers. The obtained carriers were washed with water and soap, rinsed well with distilled water, followed by 96% ethanol, left to dry, and autoclaved. Before experiments, the carriers were placed in 3 replicates on a sterile petri dish. Each disc was overlaid with 50 μL of the virus–protein solution, evenly distributed on the carrier, and left to dry for 60 min.

### 2.5. Study Design

All experiments were performed thanks to the courtesy of State Fire Service in Poland, in collaboration with Rescue and Fire Fighting Services No. 6 in Krakow, Poland. The study was conducted in 5 identical rooms of the dimensions 480 × 330 × 300 cm (W × D × H), measuring 47.52 m^3^. All possible ventilation holes were sealed, including a gap under the door to maintain the ozone concentration over time. The ozone generator was placed on the floor about 3 m distance from the table, where Petri dishes containing test discs were placed, to avoid the direct ozone flow on the carriers.

The ozone was generated by using a dielectric barrier discharge device Ulsonix Airclean 20G-Eco (Ulsonix, Expondo S.A., Zielona Góra, Poland) for 60 min prior to inserting contaminated carriers into the respective rooms. After this time, O_3_ concentration measurements were initiated in defined time intervals using a single gas detector Draeger Pac 8000 (Draeger, Draegerwerk AG & Co. KGaA, Luebeck, Germany). Dielectric barrier discharge plasma generation results in the by production of nitrogen oxides (NOx), and the concentrations were parallelly measured by using a similar single-gas device (Gasman, Crowcon Detection Instruments Ltd., Abingdon, Oxfordshire, UK). Ambient temperature and relative humidity (RH%) were measured with Wintact WT83B (Wintact, Shenzen Wintact Electronics Co., Ltd., Guangming, Shenzen, China). The air humidity was increased in two rooms by using a humidifier filled with tap water (Sterimed Eco 20, Sterimed sp. z o.o., Mińsk Mazowiecki, Poland).

Thirty-five percent hydrogen peroxide was purchased at Chempur (Piekary Śląskie, Poland) and diluted in distilled water to prepare 7.5% (*v*/*v*) solution, which was further used for fumigation.

The measured initial environmental conditions were as follows: temperature 23.0 °C and RH = 21.8%. Ozone concentration after 60 min of pretreatment in the respective units exceeded the detector measuring range, i.e., 7.3 ppm. The study design is summarized in Table 1.

### 2.6. Quantitative Carrier Tests (QCT)

MHV-overlaid carriers were placed in respective rooms saturated with ozone and/or water vapour. Petri dishes containing carriers were placed on the table. The samples in the ozone treatment study were collected at the following time intervals: 0 (immediately after drying and prior to disinfection), 15, 30, 45, 60, 90, and 120 min. With respect to H_2_O_2_ fumigation, it was performed for 30 min, with samples collected at 5, 10, 20, and 30 min. The study was performed in triplicate, and each replicate of a specific sample was collected into a separate, sterile 50 mL tube, immediately frozen in dry ice, and stored at −80 °C until further analysis.

### 2.7. Virus–PBS Solution Test

Petri dishes with the virus-PBS solution were prepared in Unit 1 and were subjected to ozonation under low RH conditions in Unit 2. The dishes were placed at different heights (on the floor, *n* = 2; on the table, *n* = 2; 15 cm under the ceiling, *n* = 2) and subjected to ozonation for 150 min (from t = 0 min, the pretreatment was not applied). The recovered virus titre was calculated as a mean (±SD) from all samples (*n* = 6) subjected to treatment.

### 2.8. Virus Infectivity Assay

The collected tubes with carriers were filled with 5 mL of DMEM, vortexed well thrice in 5 min intervals, and immediately titrated. Virus diluted in PBS was collected from Petri dishes to 1.5 mL tubes, vortexed well, and titrated. Endpoint titration was performed in sub-confluent LR7 cells seeded in 96-well plates, with an evaluation of cytopathic effect (CPE) 48 h.p.i. Infectious virus titres were expressed as fifty per cent tissue culture infective dose per millilitre (TCID_50_/_mL_) and calculated using the Reed and Muench method, as described elsewhere (Reed and Muench, 1938).

### 2.9. Statistical Analysis of Decontamination Efficacy

Virus titres were expressed as mean log_10_TCID_50_/_mL_ (±SD) from three (QCT) or six (PBS-suspended virus tests) biological replicates. The effectiveness of treatment was assessed by calculating the infectivity reduction in sanitized test carriers compared to untreated controls. Successful decontamination was defined as ≥4 log_10_ reductions in infectivity. Statistical significance between mean titres was calculated by using a two-way analysis of variance (ANOVA) with Tukey’s post hoc test, and a *p*-value of ≤0.05 was considered significant. Statistical analyses and graphical representation of the obtained results were performed using GraphPad Prism 7.0.0 (GraphPad Software, San Diego, CA, USA). The inactivation efficiency was calculated based on the difference between log-transformed titers of untreated and treated samples, and it is expressed as log reduction value (LRV).

## 3. Results

### 3.1. Environmental Conditions

After 60 min, all subsequent ozone concentration measurements during the experiments exceeded the detector measuring range, i.e., 7.3 ppm.

RH in Unit 3 and 4 reached 49.8% at 60 min further to 54.2% by the end of the study. The NOx concentration increased with time in both groups indicating efficient ozone production.

The titre of virus stock suspended in BSA was 8.25 (±0.25) log_10_TCID_50_/_mL_, and each carrier was overlaid with 50 μL of the solution; thus, they contained 6.95 (±0.25) log_10_TCID_50_/_mL_. In order to perform virus elution and quantification during the experiment, the sample was diluted in 5 mL of the medium; therefore, the actual initial titre was 6.25 (±0.25) log_10_TCID_50_/_mL_. The titre of the virus stock suspended in PBS was 6.29 (±0.25) log_10_TCID_50_/_mL_.

### 3.2. Ozone Treatment of Virus–PBS Solution

**Figure 1:**
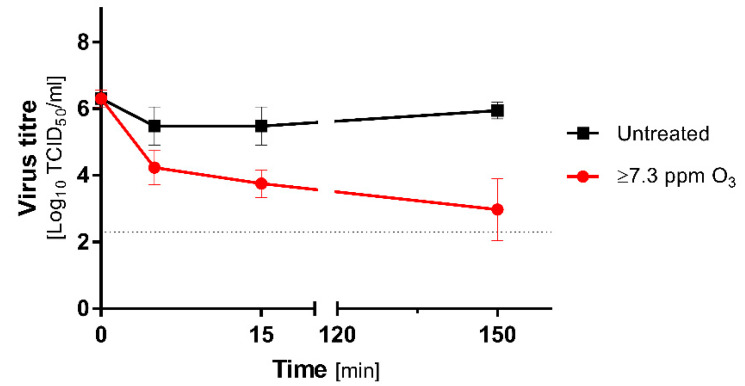
Inactivation of MHV diluted in PBS during the ozone treatment at 21.8% RH. The ozone concentration was measured from t = 60 min, and it exceeded detector measuring range, i.e., 7.3 ppm. Virus titers were calculated as a mean (±SD): *n* = 2 in the untreated group and *n* = 6 in the treated group. Red line represents the samples subjected to ozone treatment; the black line corresponds to untreated control. The horizontal dotted line represents 4 logs virucidal efficiency with respect to the initial titre.

The experiment was performed in Unit 2, and the treatment was initiated parallelly with ozone generation at t = 0 min in contrast to QCTs. The titre of the diluted virus was stable, and no significant loss of titre was observed in the untreated group during the study. The virucidal effect of ozone treatment was clearly observable from the first minutes of decontamination (Figure 1).

Regarding the initial titre, a significant loss of infectivity was demonstrated as soon as t = 5 min, and it exceeded 2 logs. The virucidal effect increased in a time-dependent manner. The MHV titre was reduced by 2.5 logs at t = 15 min. The most effective inactivation (3.3 logs) was recorded at t = 150 min. The inactivation rates between treated and untreated groups were slightly lower, but still relatively high, and reached 1.2 log (t = 5 min), 1.7 log (t = 15 min), and 3 logs (t = 150 min), respectively. The results are summarised in Table 2. Even though the treatment failed to reach the required 4 logs of titre reduction, the ozonation effectively inactivated a considerable part of the PBS-suspended virus.

### 3.3. QCT in O_3_ Treatment

#### 3.3.1. Stability of MHV on the Carriers

**Figure 2:**
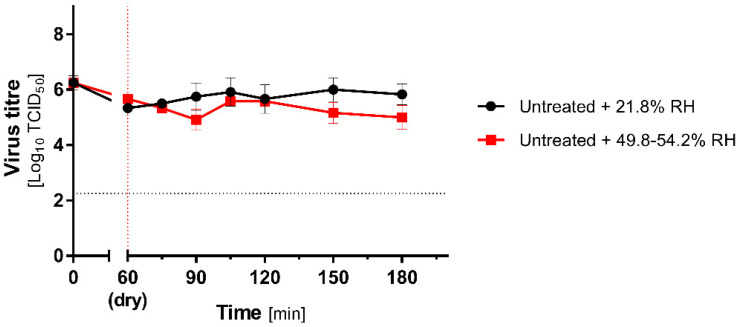
MHV stability on carriers. Stability of the MHV virus during 60 min drying on non-porous, stainless-steel surface, followed by 120 min incubation at 21.8% (low RH; black lines) or 49.8–54.2% (high RH; red lines) RH. Virus titre is calculated as mean (±SD, *n* = 3). The vertical dotted line represents the end of drying, and the horizontal dotted line represents 4 logs virucidal efficiency with respect to the initial titre.

The stability of the virus dried on the carriers was investigated at low and increased RH of untreated groups. MHV was resistant to 60 min drying and further incubation at room temperature for the next 120 min. During this time, the infectivity decreased from 6.25 (±0.25) to 5.33 (±0.14) and 5.66 (±0.14) log_10_TCID_50_/_mL_ in different groups (Figure 2). While slight differences between low and high RH groups were observed in the subsequent time points, the statistical significance (*p* < 0.05) was demonstrated only at 90, 150, and 180 min time points.

#### 3.3.2. Low RH

Low RH experiments with ozone were conducted in Unit 1 (Untreated + Low RH) and Unit 2 (O_3_ Treated + Low RH). Before the experiment, the ozone generator was initiated and operated for 60 min in order to reach a stable gas concentration. Next, the test discs were placed in the room and incubated. During the incubation, the ozone generator was operated until the end of the experiment.

**Figure 3:**
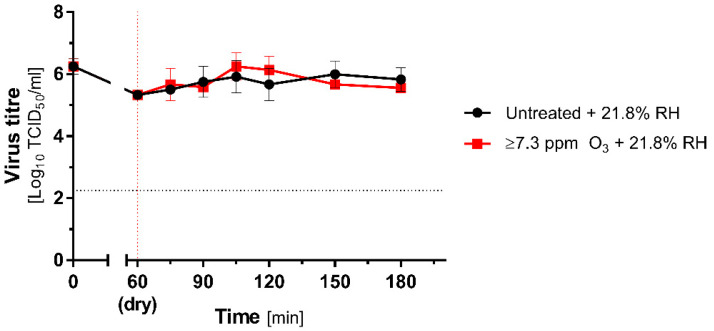
Inactivation of the coronavirus during the ozone treatment under low RH (21.8% RH). Comparison of mean (±SD, *n* = 3) titres of MHV recovered from contaminated carriers subjected to ozone treatment (≥7.3 ppm; red lines) and untreated (black lines) at 21.8% RH. Black dotted line represents the 4 logs virucidal efficiency threshold with respect to the initial titre; the red dotted line represents the beginning of O_3_ treatment.

No significant decreases in MHV titre in relation neither to the initial virus load nor to untreated group were observed during the ozone decontamination. Even after 120 min treatment, no inactivation of MHV was recorded (Figure 3).

#### 3.3.3. High RH

**Figure 4:**
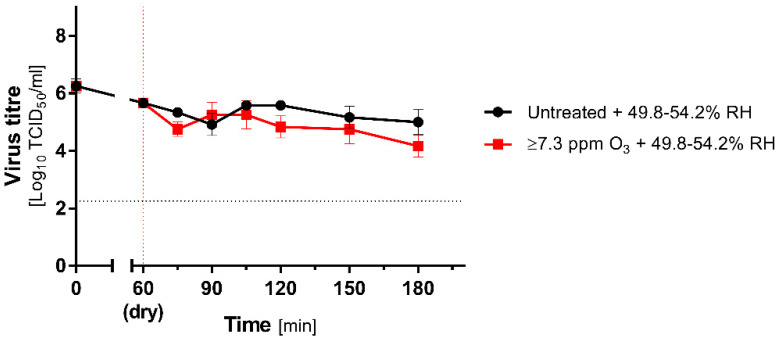
Inactivation of coronavirus during ozone treatment under high RH. Comparison of mean (±SD, *n* = 3) titres of MHV recovered from contaminated carriers subjected to ozone treatment (≥7.3 ppm, red lines) and untreated (black lines) at 49.8–54.2% RH (high RH). The vertical dotted line represents the end of drying and beginning of O_3_ treatment, and the horizontal dotted line represents 4 logs virucidal efficiency threshold with respect to the initial titre.

This part of the study was performed in Unit 3 (Untreated + High RH) and Unit 4 (O_3_ Treated + High RH). Before experiments, both rooms were subjected to 60 min vaporization with tap water, which resulted in an increase in RH from 37.7% to 49.8%. Parallelly, Room 4 was pre-treated with ozone; therefore, after 60 min, its concentration exceeded 7.3 ppm. During ozone treatment, infectivity reduction in comparison to the initial titre of undried virus control was demonstrated (Figure 4):

Here, we observed a statistically significant decrease in the virus titre, but the inactivation rate was relatively poor. At the end of the study, the titre of the recovered virus reached 4.17 (±0.32) log_10_TCID_50_/_mL_, reflecting the 2.08 log_10_TCID_50_/_mL_ reduction in the initial dose. Considering that no required reduction in the coronavirus titre was recorded (4 logs), the method may also be considered insufficient. Importantly, when the reference group (untreated) was considered instead of the t0 group, the decrease was even milder, reaching −0.83 log_10_TCID_50_/_mL_ at t = 180 min (Figure 4).

### 3.4. QCT in H_2_O_2_ Fumigation

**Figure 5:**
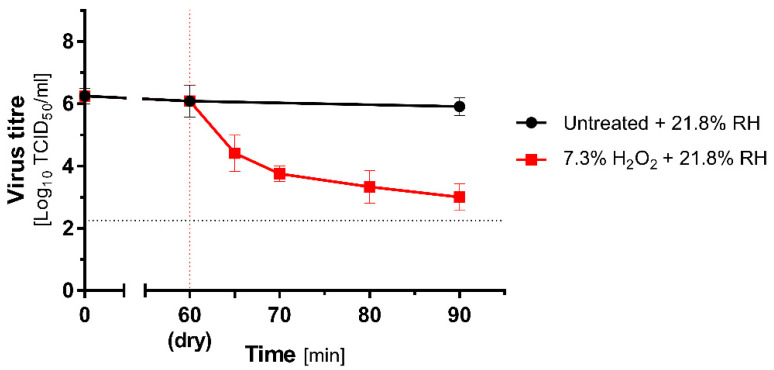
Effectiveness of H_2_O_2_ treatment on MHV dried on stainless steel carriers. Comparison of mean (±SD, *n* = 3) titres of MHV recovered from contaminated carriers subjected to hydrogen peroxide treatment (7.3%; red lines) and untreated (black lines) at 21.8% RH (low RH). The vertical dotted line represents the end of drying and beginning of H_2_O_2_ treatment, and the horizontal dotted line represents 4 logs virucidal efficiency threshold with respect to the initial titre.

An experiment with vaporized 7.5% hydrogen peroxide solution was conducted in Unit 5, with fogging intensity adjusted relative to the interior cubature according to device manufacturer guidelines. The room was not subjected to any pretreatment before the experiment, and the initial temperature and RH were the same as in the other units, i.e., 23 °C and 21.8% RH. The decontamination lasted 30 min, and the control sample at t = 30 min was collected. The drying and incubation itself did not cause a significant loss of infectivity in the untreated groups. However, a considerable decrease in virus titre was observed already at t = 5 min. A time-dependent effect was recorded for the H_2_O_2_ vaporization (Figure 5). The highest inactivation rate corresponding to 3.25 log_10_TCID_50_/_mL_ reduction compared to the initial titre was observed for t = 30 min.

The obtained results are summarized in Table 2. In general, hydrogen peroxide action was robust and caused rapid reduction in infectious virus titre by 1.5–3.3 logs under ambient conditions. For the ozone treatment, its virucidal activity was limited and dependent on the relative humidity of the air. The greatest efficiency was observed at high RH, where 53.58–85.32% reduction was noted. However, compared to the initial virus titre, the reduction was higher and oscillated between 73.9 and 99.17%.

## 4. Discussion and Conclusions

The ozone, due to its strong oxidizing properties, has been considered an effective disinfectant for years. Its biocidal activity was repeatedly demonstrated with respect to bacteria, fungi, and a wide range of viruses. Our study investigated the efficiency of gaseous ozone on the virus suspended in PBS and exposed to 150 min treatment in a real-conditions setting, with the ozone concentration exceeding 7.3 ppm at t = 60 min. The selected conditions were more robust than required to achieve successful disinfection, within the widely accepted scope of 1 g of ozone per 10 m^3^, which in our setting should be achieved in less than 15 min. We showed more than 3 logs efficiency of ozonation with regard to suspended MHV coronavirus. The obtained results stand in line with previous reports, and ozone treatment may be considered as a quite effective disinfection method for viruses suspended in liquids. Nevertheless, the inactivation rate did not fully meet international standard requirements; therefore, the method cannot be considered sufficient.

Since gaseous ozone treatment is inexpensive and easily accessible, it has become a widely used tool for the decontamination of offices, classrooms, and other public spaces during the COVID-19 pandemic. Up to this point, several studies investigated the efficiency of gaseous ozone treatment on coronavirus contaminated fomites [24,26,36,37,38,39]. However, all these experimental works were performed under strictly controlled laboratory conditions, with the application of airtight and low-volume chambers, which may not directly translate into a realistic environment [24,26,37,38,39]. To our knowledge, this is the first report investigating the efficacy of gaseous ozone treatment against SARS-CoV-2 surrogate in conditions mimicking its actual use. Biosafety issues disqualified the application of any human coronavirus; therefore, murine hepatitis virus was selected for this study. The virus is a non-pathogenic human representative of betacoronaviruses, with similar virion architecture as SARS-CoV-2, which is commonly used as a reference model for this BSL3+ pathogen [31,32,33,34,35].

Previous studies confirmed its virucidal activity against coronaviruses. Nevertheless, with respect to international standards requiring at least four orders of magnitude titre reduction, effective inactivation was confirmed only by Yano et al. [26]. It was demonstrated that 55 min/6 ppm ozone treatment reduced SARS-CoV-2 titre by 4.23 log_10_TCID_50_/_mL_ compared to initial titre, but only about 3.3 log_10_TCID_50_/_mL_ compared to untreated control [26]. In other reports, the observable reduction in infectivity oscillated between 80 and 99.8%, depending on the type of surface, humidity, ozone concentration, and treatment duration [24,37,38]. In our study, the virucidal activity of ozone treatment was limited. The greatest rate of surficial MHV inactivation was obtained at high RH after 2 h treatment with concentration >7.3 ppm, and it reduced over 2 logs of the virus in relation to initial titre. Nevertheless, ozonation is usually performed under ambient RH, for a much shorter period and without any pretreatment. These reasons, supported by our results, allow for the assumption that the virucidal efficiency of ozone is insufficient in field conditions.

The addition of interfering substances is common when assessing virucidal activity, including carrier and suspension tests [30,40]. Protein content plays a role as a virus protective agent, enhancing virus survivability even during heat-mediated decontamination [14], as interfering proteinaceous substances imitates various soiling, including human and animal biological fluids in which the virus is likely suspended. Contrary to previous publications, in our experiment, the virus was mixed with the bovine serum albumin (0.3 g/L), which probably protected virions and increased virus survival. Hence, we noted a decreased rate of virus inactivation compared to earlier reports, where additional protein content was not included. Nevertheless, an artificial saliva with mucin protein content most likely better imitates the clinical secretions; thus, such an interfering buffer may be applied in future virus stability studies. On the other hand, BSA soiling content that was deployed is widely required by various international disinfection standards; therefore, its application enables the comparison of results between various methods.

Virus survival on the surfaces also depends on environmental conditions, including temperature and relative humidity. Several studies showed that high RH conditions favour faster inactivation of surficial coronaviruses [38,41,42]. The ozone is unstable, especially when in contact with water; thus, under high RH conditions, it forms free radicals, which may improve disinfection effectivity [19,43]. In our study, at lower RH, MHV inactivation was ineffective, which stands in line with previous data. Ozone treatment was slightly more effective at high RH, particularly with respect to the initial titre. This outcome is most likely caused by two factors: lower virus stability and formation of more free radicals during ozone decomposition at higher RH. Therefore, high humidity should be required and verified during ozone treatment in order to improve its decontamination effectiveness. Nevertheless, the presence of high-water content in the air during ozone generation by dielectric barrier discharge has a serious disadvantage since it results in decomposition of nitrogen and formation of nitrogen radicals. Subsequent chemical reactions may result in the by-production of nitric acid, acting as a caustic and corrosive agent for certain materials [44].

One may speculate that higher ozone concentrations may be used for the disinfection of coronavirus-contaminated spaces. In the controlled laboratory environment, this is relatively easy to achieve, but in the real-world setting it would require external supply of concentrated oxygen. This would drastically increase the cost and technical requirements of the procedure. Furthermore, higher ozone concentrations may be corrosive, especially under high relative humidity, and the risk for humans and animals should also be considered. Nonetheless, further laboratory studies would likely provide valuable results for future applications.

Room decontamination using vaporized hydrogen peroxide has been known for years as an efficient sanitation method; thus, it is widely used in various areas [43]. Therefore, in addition to O_3_, we also investigated the virucidal efficiency of hydrogen peroxide treatment in a similar experimental setup under low RH conditions. Even though a much shorter time was applied, the effect was observed in a time-dependent manner. Half-hour treatment resulted in virus titre reduction by 3 logs compared to untreated control and by 3.3 logs to the initial titre. Although these results do not fully fulfil the requirements of the standard of 4 logs inactivation, hydrogen peroxide virucidal activity is inarguably far much robust than gaseous ozone in similar conditions. Other studies demonstrated that increased RH is a favourable factor for H_2_O_2_ treatment effectivity [45]. Nevertheless, due to space limitations, we were unable to compare the efficiency of hydrogen peroxide vaporization between ambient and increased RH. It should be highlighted that H_2_O_2_ fumigation itself causes RH increase, but pretreatment with water vapour may be required to provide the most efficacious decontamination.

The main aim of this study was to evaluate the efficiency of gaseous ozone treatment in inactivation of surficial SARS-CoV-2 surrogate under field conditions with application of low and high RH. In low RH, the obtained virucidal activity of the decontamination oscillated between 0 and 0.9 logs in relation to initial titre, revealing the insufficiency of applied method in the context of international disinfection standards. In high RH, the efficiency was slightly better and reached 0.6 up to 2.1 logs, thus indicating favourable influence of humidity on virus inactivation during ozone treatment. Nevertheless, having regard to required 4 logs efficiency, the method may still be considered ineffective. An additional test of the ozonation was performed using PBS-suspended virus. In this case, we demonstrated that the performance increased up to more than 3 logs, even at low RH and shorter time applied in comparison to carrier tests. However, the obtained efficacy was still insufficient to demonstrate adequate virucidal effect. The last part of the study was committed to investigating the effectivity of H_2_O_2_ fumigation by QCT in similar experimental setups. The results showed high virus inactivation rate from the very first minutes of the treatment and more than 3.3 logs efficiency after 30 min of fumigation. The treatment showed the best efficacy out of the tested methods.

Due to biosafety issues and the field nature of this study, the use of human pathogens was not feasible. However, it is common to use low-pathogenic surrogate viruses as models, especially in studies addressing environmental stability and decontamination efficacy [33,34,35,46]. While such an approach has its drawback, it allows for real-world study that is superior to artificial laboratory conditions. Nevertheless, the selection of surrogate virus should be supported by the genetic similarity and physicochemical properties with the original virus [46]. In our study, MHV was selected as the best fit-for-purpose model since it belongs to the same family (betacoronaviruses) and shows similar shape, size (80–100 nm), and virion structural architecture as SARS-CoV-2 [47,48].

Contrarily to previous studies, we showed that the most popular method of gaseous ozone generation through corona barrier discharge is highly insufficient under field conditions in relation to surficial viruses. Regardless of the applied humidity level, it cannot be treated as a universal solution preventing indirect transmission of infectious viral diseases. However, the method still has a great potential as a complementary tool, which may be useful for inactivating residual viruses after basic cleaning. On the other hand, hydrogen peroxide treatment showed much better performance in QCTs than ozonation in similar experimental conditions, indicating that it presents as a better solution for gaseous decontamination. Nevertheless, all tested methods have certain disadvantages and limitations; therefore, its application needs to be performed responsibly by well trained staff because any failure in decontamination process poses a relevant risk for human health, while providing a false sense of security.

## Figures and Tables

**Table 1:** Units in the study. Description of the environmental conditions applied in respective study units. Pretreatment was applied for 60 min and followed by 120 min (ozone) or 30 min (hydrogen peroxide) of treatment.

Unit Number	Unit Name *	Pretreatment	Conditions after Pretreatment	Treatment	Conditions after Treatment
**1**	Untreated + Low RH	No	RH = 21.8%	No	RH = 21.8%
**2**	Treated O_3_ + Low RH	60 min O_3_	RH = 21.8% O_3_ > 7.3 ppm	120 min O_3_	RH = 21.8% O_3_ > 7.3 ppm
**3**	Untreated + High RH	60 min humidification	RH = 49.8%	120 min humidification	RH = 54.2%
**4**	Treated O_3_ + High RH	60 min humidification 60 min O_3_	RH = 49.8% O_3_ > 7.3 ppm	120 min O_3_120 min humidification	RH = 54.2% O_3_ > 7.3 ppm
**5**	Treated H_2_O_2_ + Low RH	No	RH = 21.8%	30 min H_2_O_2_	RH = n/a H_2_O_2_ = n/a

* With respect to the Figures.

**Table 2:** Reduction in the MHV titres after fumigation. The results are presented as LRV and denote the reduction in the viral titre in treated samples, as compared with the initial levels. Plus signs represent samples, where an untreated sample showed a lower titre than a treated one. Asterisks represent statistical significance: (*) *p* < 0.05; (**) *p* < 0.01; (***) *p* < 0.001; non-significant reduction is not marked. The measured values of RH were as follows: 21.8% RH is marked as low, and 48.8–54.2% RH is marked as high RH.

**QCTs**	**LRV** **between Untreated and Treated Samples**					**LRV** **between the Initial Titre and Treated Samples**				
	**Ozone**			**Hydrogen Peroxide**		**Ozone**			**Hydrogen Peroxide**	
	**Timepoint (min)**	**Low RH**	**High RH**	**Timepoint (min)**	**Low RH**	**Timepoint (min)**	**Low RH**	**High RH**	**Timepoint (min)**	**Low RH**
	**0**	0.0	0.0	**0**	0.0	**0**	0.0	0.0	**0**	0.0
	**60**	0.0	0.0	**60**	0.0	**60**	0.9	0.6	**60**	0.2
	**75**	+0.2	0.6	**65**	1.5 (***)	**75**	0.6	1.5 (***)	**65**	1.8 (***)
	**90**	0.2	+0.3	**70**	2.2 (***)	**90**	0.7	1.0 (*)	**70**	2.5 (***)
	**105**	+0.3	0.3	**80**	2.6 (***)	**105**	0.0	1.0 (*)	**80**	2.9 (***)
	**120**	+0.5	0.8	**90**	2.9 (***)	**120**	0.1	1.4 (***)	**90**	3.3 (***)
	**150**	0.3	0.4			**150**	0.6	1.5 (***)		
	**180**	0.3	0.8 (*)			**180**	0.7	2.1 (***)		
**Virus Suspension**	**0**	0.0	n/a	**n/a**	n/a	**0**	0.0	n/a	**n/a**	n/a
	**5**	1.2	n/a	**n/a**	n/a	**5**	2.1 (***)	n/a	**n/a**	n/a
	**15**	1.7 (**)	n/a	**n/a**	n/a	**15**	2.5 (***)	n/a	**n/a**	n/a
	**150**	3.0 (***)	n/a	**n/a**	n/a	**150**	3.3 (***)	n/a	**n/a**	n/a

## Data Availability

The data presented in this study are available in article.
